# Solid-State Emissive Metallo-Supramolecular Assemblies of Quinoline-Based Acyl Hydrazone

**DOI:** 10.3390/s20030600

**Published:** 2020-01-21

**Authors:** Hye Jin Cho, TaeWoo Kim, Hyunwoo Kim, Changsik Song

**Affiliations:** Department of Chemistry, Sungkyunkwan University, 2066 Seobu-ro, Jangan-gu, Suwon-si, Gyeonggi-do 16419, Korea; chj9420@skku.edu (H.J.C.); xodntodn@gmail.com (T.K.); khw2651@naver.com (H.K.)

**Keywords:** hydrazone, metallo-supramolecular assembly, solid-state emission

## Abstract

Development of fluorescence-based sensory materials for metal elements is currently in the mainstream of research due to the simplicity and usability of fluorescence as a method of detection. Herein, we report a novel “*bis*”-quinoline-based acyl hydrazone—named bQH that could be synthesized by a facile, low-cost method through simple condensation of hydrazide with an aldehyde. This acyl hydrazone showed emissive properties through Zn selective binding, especially in its solid-state, as shown by experiments such as UV–Vis, photoluminescence (PL), nuclear magnetic resonance (NMR), and inductively-coupled plasma-optical emission spectroscopies (ICP-OES), and energy-dispersive X-ray spectroscopy (EDS) mapping. The binding modes in which bQH coordinates to Zn^2+^ was proved to consist of two modes, 1:1 and 1:2 (bQH:Zn^2+^), where the binding mode was controlled by the Zn^2+^ ion content. Under the 1:1 binding mode, bQH-Zn^2+^ complexes formed a polymeric array through the metallo-supramolecular assembly. The resulting bQH-Zn^2+^ complex maintained its fluorescence in solid-state and exhibited excellent fluorescence intensity as compared to the previously reported quinoline-based acyl hydrazone derivative (mQH).

## 1. Introduction

The development of artificial probes to detect metal ions is of great interest because of the tendency of metal ions to act as typical pollutants and essential trace elements in biological systems. The detection of the different species of metal ions by spectroscopic changes—absorption or emission—has been under investigation for the last few decades [1,2]. Based on its simplicity, precise and instantaneous response, and nondestructive properties, chemo-sensors capable of fluorescence detection are becoming more popular compared to conventional analytical methods [3,4].

Zinc is the second most abundant transition metal ion in the human body that plays important metabolic roles, including those in neurological signaling and enzymatic reactions, cell growth, protein and DNA synthesis, and immune function [5,6]. Several artificial receptors for Zn ion have been developed based on quinoline [7,8,9,10,11,12,13], coumarin [14,15,16,17,18,19,20], benzoxazole [21,22,23,24], BODIPY [25,26], BINOL [27,28,29], fluorescein [30,31,32], and rhodamine [33,34,35,36,37] fluorophores. 

Hydrazone, a well-known functional group often present in stimuli-responsive materials, is a promising candidate for Zn ion sensing [38]. Several studies have reported hydrazone-based compounds that can bind metal ions. Various metal ions, including Al^3+^, Cu^2+^, Ru^2+^, Pd^2+^, and Pt^2+^, can form a hydrazone-metal complex, and thus, may be applied in photochromic systems [39], molecular motors [40,41], proton transfer systems [42,43], bioactive materials [44,45,46,47], etc. Acyl hydrazone derivatives are an especially popular type of hydrazone derivatives for metal ion binding. Among them, hydrazone derivatives with quinoline [48,49,50], the 3-hydroxy-2-naphthoyl group [51,52], thiophene [53], pyrazole [54], or fluorescein [55] have been reported to bind Zn ions and exhibit fluorescence. Although a variety of hydrazone derivatives are known to selectively bind to the Zn ion, they have seldom been expanded to form a supramolecular assembly [56,57].

**Figure 1:**
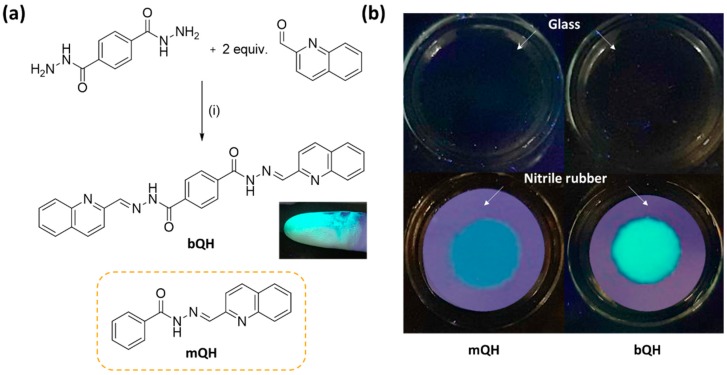
(**a**) Synthesis of *bis*-quinoline-based acylhydrazone (bQH) and the structure of mono-quinoline acyl hydrazone (mQH). Reaction conditions: (i) Few drops of AcOH, MeOH, rt, 8 h, 88%. Inset: Emission of bOH on nitrile rubber gloves under 365 nm ultraviolet (UV) lamp. (**b**) Images of bQH and mQH (2.5 × 10^−4^ M in dimethyl sulfoxide (DMSO), 20 μL) dropcasted on glass (upper) and nitrile rubber (bottom), under 365 nm UV lamp.

Herein, we have designed and synthesized a novel quinoline-based acyl hydrazone, bQH (Figure 1a), that is capable of forming a metallo-supramolecular assembly. It can simply be synthesized by reacting terephthalic dihydrazide with two equivalents of 2-quinolinecarboxaldehyde under ambient conditions. Interestingly, bQH exhibited a bright green emission when its solution was brought into contact with nitrile rubber gloves (Figure 1a, inset). In comparison to previous reports on hydrazone-based Zn sensing, which dealt with its solution phase, bQH maintained its emission even in solid-state by binding Zn^2+^ directly on its surface. Further investigations have been employed here to study the emission of bQH, and it has finally been proven that the emission observed on nitrile rubber gloves was due to the selective formation of emissive hydrazone-Zn ion complex. The selectivity of bQH toward Zn was also confirmed by UV–Vis absorption and emission spectra of bQH solutions with various metal ions. Combined with the relatively weak binding strength of bQH toward Zn ion, the two binding sites of bQH enabled it to have two binding modes. It was observed that bQH first formed a polymeric array of the metallo-assembly under the appropriate concentration of Zn^2+^. Once the Zn ion fraction increased, the supramolecular polymer, i.e., bQH-Zn complex, disassembled into bQH-Zn_2_; this sequence of events was confirmed by a continuous variation method (Job plot) and ^1^H-NMR titration.

## 2. Materials and Methods

### 2.1. General

All the chemicals were purchased from commercial sources, Sigma-Aldrich (Seoul, Korea), Alfa Aesar (Seoul, Korea), TCI (Tokyo, Japan), Acros Organics (Geel, Belgium), or Samchun Chemical (Seoul, Korea). The chemicals were used as received, without further purification. All reactions were done under an inert atmosphere using standard Schlenk techniques. The nature of synthesized materials was confirmed using nuclear magnetic resonance (NMR) spectroscopy (500 MHz, Bruker); chemical shifts are reported in ppm (δ) relative to the DMSO-*d*_6_ solvent residual peak (δ 2.50) and coupling constants (*J*) are expressed in Hz. Deuterated solvents were purchased from Cambridge Isotope Laboratories (Tweksbury, MA, USA). High-resolution mass spectra (HRMS) were obtained on a JEOL JMS-700 using 3-nitrobenzyl alcohol as a matrix. UV–Vis absorption measurements were obtained on a UV-1800 (Shimadzu) spectrophotometer under ambient conditions (room temperature, air) with a 1.0 cm quartz cell. Photoluminescence (PL) measurements were performed on a FluoroMate Fs-2 (Scinco). Scanning electron microscope (SEM) image and energy dispersive X-ray spectroscopy (EDS) mapping spectrum were obtained by using FE-SEM JEOL-7100 after vacuum-drying of the piece of nitrile rubber on the grid.

### 2.2. Synthesis of (E)-N′-(quinolin-2-ylmethylene)benzohydrazide (mQH)

The synthesis of mQH was carried out as noted in the previous literature [58]. In a flask, 2-quinolinecarboxaldehyde (0.157 g, 1 mmol) was added to a suspension of terephthalic dihydrazide (0.136 g, 1 mmol) in 25 mL of ethanol and mixed for 8 h under room temperature. The reaction mixture was then poured into water and filtered with water under room temperature and dried overnight in a 60 °C oven. The obtained crude solid was dissolved in dimethylformamide (DMF) under room temperature, which was recrystallized from H_2_O (ratio of DMF:H_2_O = 1:9) to give the products in 96% yield (yellowish solid, 0.264 g). ^1^H NMR (500 MHz, DMSO-*d*_6_) δ 12.21 (s, 1 H), 8.63 (s, 1 H), 8.44 (d, *J* = 8.4 Hz, 1 H), 8.14 (d, *J* = 8.4 Hz, 1 H), 8.05 (d, *J* = 8.4 Hz, 1 H), 8.02 (d, *J* = 8.0 Hz, 1 H), 7.96 (d, *J* = 7.2 Hz, 2 H), 7.80 (t, *J* = 7.5 Hz, 1 H), 7.64 (t, *J* = 7.2 Hz, 2 H), 7.57 (t, *J* = 7.4 Hz, 2 H) ^13^C NMR (125 MHz, DMSO-*d*_6_) δ 163.53, 153.81, 147.96, 147.37, 136.77, 133.20, 132.05, 130.10, 128.91, 128.59, 128.03, 127.92, 127.78, 127.34, 117.51.

### 2.3. Synthesis of (N′1E,N′4E)-N′1,N′4-bis(isoquinolin-3-ylmethylene)terephthalohydrazide (bQH) [59]

In a flask, 2-quinolinecarboxaldehyde (2 mmol) was added to a suspension of terephthalic dihydrazide (1 mmol) in 20 mL of methanol and mixed for 8 h in the presence of 100 μL of glacial acetic acid. The reaction mixture was then poured into water and filtered with water under room temperature and dried overnight in a 60 °C oven. The crude solid thus obtained was dissolved in DMF under room temperature, which was recrystallized from H_2_O (ratio of DMF:H_2_O = 1:9) to obtain a product yield of 88% (yellowish solid, 0.415 g). ^1^H NMR (500 MHz, DMSO-*d*_6_) δ 12.36 (s, 2 H), 8.65 (s, 2 H), 8.47–8.45 (d, *J* = 8.6 Hz, 2 H), 8.17–8.15 (d, *J* = 8.7 Hz, 2 H), 8.13 (s, 4 H), 8.08–8.06 (d, *J* = 8.4 Hz, 2 H), 8.04–8.03 (d, *J* = 7.9 Hz, 2 H), 7.83–7.80 (t, *J* = 7.7 Hz, 2 H), 7.67–7.65 (t, *J* = 7.6 Hz, 2 H). ^13^C NMR (125 MHz, DMSO-*d*_6_) δ 162.78, 153.70, 148.52, 147.39, 136.84, 136.12, 130.15, 128.95, 128.05, 127.98, 127.42, 117.55. One peak in quinoline seemed to overlap with the peak of core benzene at 128.05 ppm. MS (HRMS): M/z calculated for C_28_H_21_N_6_O_2_ [M+H]^+^ 473.1726; found: 473.1726.

### 2.4. Comparison of bQH and mQH on Different Substrates

A glass slide and a piece of nitrile rubber was prepared, and solutions of bQH and mQH (2.5 × 10^−4^ M) were dropped on the surfaces. After drying under vacuum for 12 h, a total of four samples were compared under a 365 nm UV lamp.

### 2.5. Metal Screening of bQH

Stock solution of bQH (5.0 × 10^−3^ M in DMSO) was added to the stock solutions of metal perchlorates (5.0 × 10^−3^ M in DMSO) and tetrabutylammonium cyanide (TBA-CN) (5.0 × 10^−3^ M in DMSO) in a ratio of bQH:metal perchlorate:TBA-CN = 1:4:4. The bQH solution was diluted to a concentration of 5.0 × 10^−6^ M, and added to the same stock solutions of metal perchlorates and TBA-CN in the same ratios to obtain another fluorescence spectra.

### 2.6. Absorption and Emission Measurements

A solution of bQH in DMSO (5.0 × 10^−6^ M) was prepared by diluting the stock solution (5.0 × 10^−3^ M). Zinc solutions with various anions and TBA-CN were prepared as the stock solutions (5.0 × 10^−3^ M) and were added to the desired equivalents noted in each figure. The same samples that were used in absorption measurements were used in emission measurements. For the emission spectra obtained from samples of high concentration (absorption > 0.1), a correction for the inner filter effect was performed [60].

### 2.7. Inductively Coupled Plasma Optical Emission Spectroscopy (ICP-OES) Measurement

The bQH solution (6.0 × 10^−3^ M in DMSO) was dropped on a piece of nitrile rubber and immediately turned into yellow color, so the solution was collected right after it contacted with the nitrile rubber. Concentrations of Zn(II) in the resulting solution was measured by ICP-OES (Varian) at 213.857 nm.

### 2.8. UV–Vis Spectroscopic Titration of bQH with Zn(CN)_2_

The bQH solution (1.0 × 10^−5^ M in DMSO) was added with the stock solution of Zn(CN)_2_ in DMSO (2.0 × 10^−3^ M) from 0.0 to 50 equivalents. After having an enough time to form equilibrium states (approximately up to 10 min), the absorption and emission spectra were obtained. 

### 2.9. Fluorescence Quantum Yield (FQY) Measurements

bQH solutions are the same samples used in UV–Vis spectroscopic titration. The two mQH solutions (1.5 × 10^−5^ M in DMSO, respectively) were added with the stock solution of Zn(CN)_2_ in DMSO (2.0 × 10^−3^ M) in two different equivalents of 1.0 and 5.0. After having enough time to form equilibrium states (approximately up to 10 min), the absorption and emission spectra were obtained. The relative FQY values were estimated using the absorption and emission spectra as noted in the previous literature [61].

### 2.10. ^1^H-NMR Study on bQH-Zn^2+^ Binding Modes

Solutions of bQH in DMSO-*d*_6_ (2.0 × 10^−2^ M) were prepared and 2.0 equivalents of tetraethylammonium hydroxide (TEA-OH) was added, prepared as the stock solution (5.0 × 10^−1^ M in DMSO-*d*_6_). Zinc perchlorate, also, was prepared as the stock solution (5.0 × 10^−1^ M in DMSO-*d*_6_) and used to the desired equivalents.

## 3. Results and Discussion

As mentioned above, the desired molecule “*bis*”-quinoline-based acyl hydrazone (bQH) was synthesized from the reaction of terephthalic dihydrazide and 2-quinolinecarboxaldehyde to obtain a good yield (88%) (Figure 1a). The identity of bQH thus synthesized was confirmed using nuclear magnetic resonance (NMR) spectroscopy and high-resolution mass spectroscopy (HRMS).

Bright green fluorescence of bQH was first observed when nitrile rubber (acrylonitrile butadiene rubber, NBR) gloves were stained with it. We also employed various substrates including glass, polyacrylonitrile (PAN), latex gloves, Teflon, and nylon filter paper. Among all the other substrates, bQH only emitted fluorescence on the nitrile rubber gloves (Figure S1). To unveil the reason of this solid-state emission, energy dispersive X-ray spectroscopy (EDS) was conducted with NBR gloves (Figure S2). According to the EDS spectrum, various metal cations were dispersed on the surface of the NBR gloves. Hence, we hypothesized that bQH binds with metal ions extracted from the surface of NBR gloves to produce an emissive compound.

Metal binding of quinoline-based acyl hydrazone was reported previously with derivatives of half-structured (*E*)-*N*′-(isoquinolin-3-ylmethylene)benzohydrazide (mQH). To observe the similarities between mQH and bQH, solutions of each compound were drop-casted on the surface of glass and NBR gloves (Figure 1b). Unlike the bQH, which was emissive on the NBR gloves, mQH seemed to be less or almost nonemissive on both surfaces.

**Figure 2:**
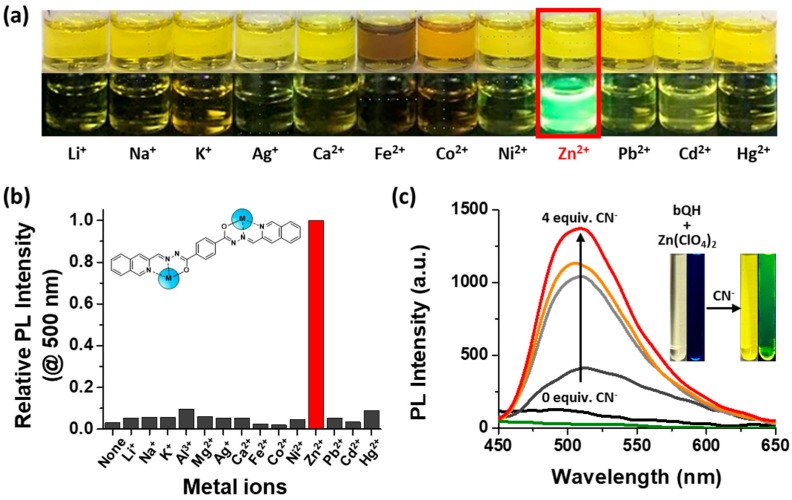
Selective binding of bQH to Zn^2+^ ion. (**a**) Images of bQH solutions (5.0 × 10^−3^ M in DMSO) mixed with an excess amount of each metal ion (upper). The same samples were placed under a 365 nm UV lamp (bottom). (**b**) Comparison of the photoluminescence (PL) intensity of bQH (5.0 × 10^−6^ M in DMSO) with 2.0 equivalents of metal ions at λ = 500 nm, presented relative to the PL intensity of bQH with Zn^2+^ ion. (**c**) Increment in PL intensity of bQH (5.0 × 10^−6^ M) with 4.0 equivalents of Zn(ClO_4_)_2_ by addition of tetrabutylammonium cyanide (TBA-CN) up to 4.0 equivalents in DMSO (measurement conditions: Lamp voltage of 500 V, 5 nm slit width, and λ_ex_ = 350 nm). The green line is the emission spectrum of Zn(ClO_4_)_2_ (2.0 × 10^−5^ M in DMSO). Inset: Color change (left) and PL increment (right) of bQH solution mixed with Zn(ClO_4_)_2_ achieved by adding CN^−^.

Several solutions with metal ions were prepared to determine whether bQH possessed metal sensing property. In view of the EDS data, Li^+^, Na^+^, K^+^, Al^3+^, Mg^2+^, Ag^+^, Ca^2+^, Fe^2+^, Co^2+^, Ni^2+^, Zn^2+^, Pb^2+^, Cd^2+^, and Hg^2+^ solutions were prepared (Figure 2a and Figure S3a). The bQH solution with Zn^2+^ ion showed a bright green fluorescence under UV lamp (λ = 365 nm). At first, it seemed that the Al^3+^ and Mg^2+^ could also be detected by bQH because they also emitted fluorescence in the presence of bQH in high concentration (5.0 × 10^−3^ M). However, the Zn specific metal binding property of bQH was confirmed by PL spectra. The metal ion stock solution (5.0 × 10^−3^ M) was added to the solution of bQH (5.0 × 10^−6^ M) in the amount of two equivalents in DMSO. The PL spectra of metal-combined bQH solution were measured under the same conditions (Figure S3b). A clear specificity of bQH toward Zn^2+^ was observed, showing strong emission, while the others were almost nonemissive with the relative PL intensity of 10% (at λ = 500 nm). In addition, bQH bind Zn2+ selectively in the presence of other competitive metal ions such as Cd^2+^, Ag^+^, or Li^+^ (Figure S4). bQH appeared to bind Ag^+^ or Li^+^ very little, but proved to be able to coordinate to Cd^2+^ to form the bQH-Cd^2+^ complex, resulting in the absorption change. Interestingly however, the solution of bQH-Cd^2+^ was almost nonemissive. Both Cd^2+^ and Zn^2+^ can form the bQH-M^2+^ complex, but bQH seemed to bind Zn^2+^ more likely than Cd^2+^. By the successive addition of Zn^2+^, the solution became emissive, which we attributed to the fact that Cd^2+^ in bQH-Cd^2+^ was replaced to Zn^2+^.

Further, we investigated the anion effect on the metal binding capacity of bQH. We proposed that the emission of bQH may be derived from the bQH-metal complex in which complex formation occurs in two steps: (1) Deprotonation followed by (2) metal ion binding. Deprotonation alone cannot make bQH emissive (Figure S5). The emission of the bQH-Zn^2+^ metal complex (Figure 2a,b) could be observed only after the addition of Zn^2+^ ion accompanied by a change in the absorption spectra (Figure S6). The reason behind the nonemissivity bQH and Zn^2+^ mixture was the nonbasic bulky ClO_4_^−^ anion. The bQH solution with Zn(ClO_4_)_2_ started to be emissive immediately with the addition of CN^−^ anion, which is basic enough to deprotonate the acidic proton of bQH (Figure 2c). A further experiment on the anion effect was performed using Zn^2+^ with various anions—CN^−^, SO_4_^2−^, Br^−^, NO_3_^−^, ClO_4_^−^. The nonbasic anions (Br^−^, NO_3_^−^, ClO_4_^−^) did not change the absorption spectra, while the basic anions led to an increasing peak at 420 nm with increasing PL intensity (Figures S7 and S8). According to the previous literature, the pK_a_ of acyl hydrazone N–H ranges from 8.8 to 11.3 in aqueous solution [62]. The pH value of bQH solution (5.0 × 10^−6^ M in DMSO) was measured to be 8.0 using a pH meter. Assuming that the bQH is a weak acid, the pK_a_ value of bQH estimated from the pH value was 10.7. Therefore, only the anions which have pK_a_ of its conjugate acid greater than 10.7 could deprotonate bQH.

Furthermore, bQH seemed to have a much higher quantum yield as compared to monomeric mQH. The results of the addition of Zn(CN)_2_ to bQH and mQH were compared (Figure S9). Upon addition of Zn(CN)_2_, both bQH and mQH showed similar changes in their absorption spectra, a decrease in peak intensity at 320 nm and an increase at 420 nm. However, the bQH with four equivalents of Zn(CN)_2_ exhibited more intense fluorescence than the mQH, with the intensity being almost 10 times higher. This is a remarkable increment in PL intensity which could be explained only by the higher quantum yield of bQH, even if we considered that bQH had double the amount of fluorophores as compared to mQH.

The Zn^2+^ ion sensing ability of bQH was once again confirmed by inductively coupled plasma-optical emission spectroscopy (ICP-OES) (Figure S10). The stock solution of bQH (6.0 × 10^−3^ M) was dropped on a piece of NBR gloves, and the concentration of Zn^2+^ ion contained in the bQH solution was measured. According to the resulting data, approximately 20.3 ppm of Zn^2+^ was extracted from the surface of the NBR gloves.

**Figure 3:**
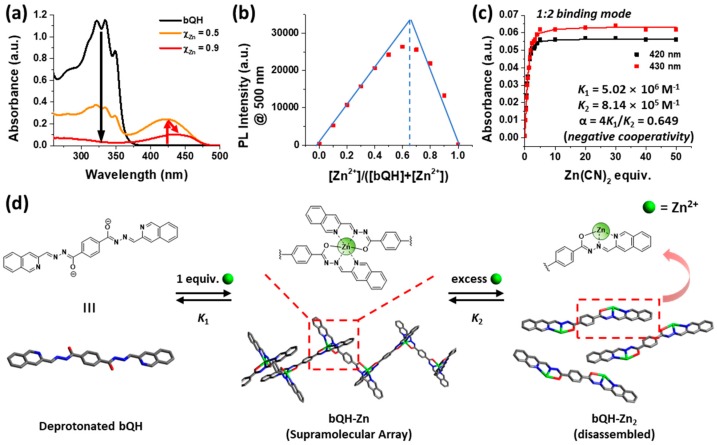
Study of the binding mechanism with ultraviolet-visible (UV–Vis) and PL spectroscopy. (**a**) The absorption spectra of a solution of bQH (1.0 × 10^−5^ M in DMSO) with increasing ratio of Zn^2+^ ion performed for the Job’s plot of bQH-Zn^2+^ ([bQH] + [Zn^2+^] = 2.0 × 10^−5^ M). (**b**) Corresponding Job’s analysis of bQH-Zn^2+^ with PL data. (**c**) The nonlinear regression analysis of absorption titration at 420 (black) and 430 nm (red) with the binding mode 1:2. (**d**) Schematic representation of the binding mechanism of bQH-Zn^2+^. The coordination occupied by any solvent molecules or ligands have been omitted for clarity.

Further in-depth investigation of the binding mechanism of bQH toward Zn^2+^ ion was performed using a continuous variation method (Job plot). Before the Job’s analysis, an unexpected change in UV–Vis absorption spectra was observed (Figure 3a and Figure S11a). During the measurement, we increased the mole fraction of Zn^2+^ (χ_Zn_); the peak at 420 nm arose as observed before because of the formation of the bQH-Zn^2+^ complex from bQH after deprotonation. A decrease in the corresponding absorption band was observed when χ_Zn_ > 0.5 and simultaneously, red shifting of the absorption band began, which indicated that at least two different species participated in the Zn^2+^ binding of bQH.

Considering the above, Job’s analysis was conducted with the PL spectra (Figure S11b). The PL intensity increased initially and then decreased after a certain point, which was the same as the UV–Vis absorption spectra (Figure 3b). The resulting Job plot exhibited a deviation from the normal triangular shape into hyperboles. Hyperbole-shaped Job plots usually appear when the binding constants are relatively small. In this case, the hyperbole-shaped plot could indicate the existence of HnGm (H: Host and G: Guest molecule; in our system, H:bQH and G:Zn^2+^) as well in several stoichiometries. The emission maximum was observed at χ_Zn_ ≈ 0.55~0.75, suggesting that both 1:1 and 1:2 binding modes could be present.

We also analyzed the binding modes of bQH toward Zn^2+^, using a UV–Vis spectroscopic titration (Figure 3c and Figure S12). Two wavelengths of 420 and 430 nm were selected to perform nonlinear regression analysis, which are the absorption maximum wavelengths of bQH-Zn and bQH-Zn_2_, respectively. Using the fitting program for 1:1 and 1:2 binding mode [63], we could obtain more reliable stepwise (thermodynamic) binding constants *K*_1_ and *K*_2_ from 1:2 binding mode (see Supplementary Materials, Table S2). The quality of fitting results was compared with calculated “cov_fit_” values. The covariance of the fit, “cov_fit_”, is a parameter which represents how the data fits well to the given plot. The lower the value, the better the model explains datasets. The *K*_1_ was estimated to be 5.02 × 10^6^ M^−1^ while the *K*_2_ was 8.14 × 10^5^ M^−1^, which are in the range of previously reported *K* values of similar chelators from 1.00 × 10^4.75^ to 1.00 × 10^9.6^ [48,49,59]. The fitting results indicated negative cooperativity (α = 4 *K*_2_/*K*_1_ < 1), meaning that bQH favors 1:1 binding (i.e., supramolecular assembly) than 1:2 binding by addition of Zn(CN)_2_. In addition, the limit of detection (LOD) and limit of quantitation (LOQ) values of bQH toward Zn(CN)_2_ were estimated to be 73.8 and 246 nM, respectively (Figure S12d). The detection limit of precedent *N*-acylhydrazone-based Zn sensors ranged from few to few tenths of nM [53,64,65,66], thus our result seems to be a reasonable value. It should be noted that bQH appears to work well for Zn^2+^ sensing in the solid state.

It has been known that two molecules of a quinoline-acyl hydrazone ligand can form an octahedral-type bond to Zn^2+^. For bQH, two quinoline moieties were connected with a rigid benzene ring, which made it impossible to allow intra-molecular binding of the two quinolines to the same Zn^2+^ ion. Thus, we hypothesized that upon addition of one equivalent of Zn^2+^ per bQH (i.e., two parts of quinoline moiety), inter-molecular binding of two quinoline moieties would form supramolecular polymers (metallo-supramolecular assembly). Further addition of Zn^2+^ would disassemble the metallo-supramolecular polymers into bQH-Zn_2_ complexes with extra coordination of solvent molecules (Figure 3d). Interestingly, supramolecular polymeric bQH-Zn and monomeric bQH-Zn_2_ complexes seemed to be emissive, even in the solid states.

The fluorescence quantum yield (FQY) measurements were performed to compare the emission properties of bQH-Zn^2+^ complexes precisely. From the emission spectra obtained during the UV–Vis spectroscopic titration, FQY values of bQH-Zn and bQH-Zn_2_ were estimated and those for Zn^2+^ bound complexes of mQH were also measured (see Supplementary Materials, Table S3) [61]. Since isolating the pure compounds was difficult, each complex were prepared by mixing the solutions of QH with two different Zn(CN)_2_ equivalents of 1.0 and 5.0. We assumed the desired form of complexes would be the major compound in the solutions. Although all the obtained FQY values were very low (~1.00%), a significant increase in emission of bQH upon binding to Zn^2+^ enabled its detection. In addition, we observed an increase 2–3 folds in FQY values of bQH-Zn^2+^ complexes compared to mQH-Zn^2+^.

**Figure 4:**
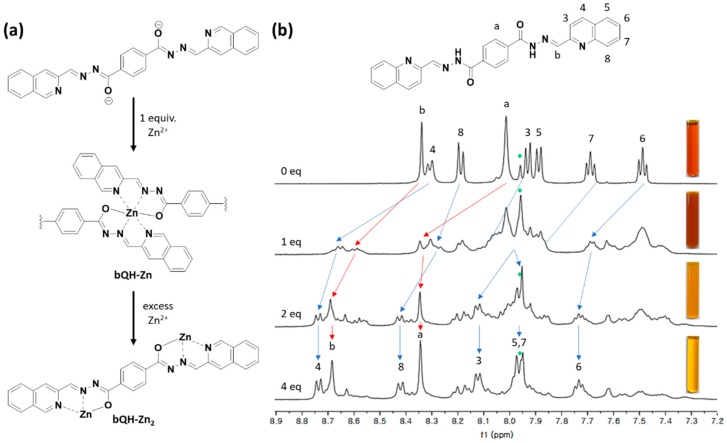
(**a**) Schematic representation of changes in deprotonated bQH followed by addition of the Zn^2+^ ion. (**b**) Partial ^1^H-NMR (500 MHz) of bQH (2.0 × 10^−2^ M) in DMSO-*d*_6_ with 2 equivalents of tetraethylammonium hydroxide, by adding up to 4 equivalents of Zn(ClO_4_)_2_. The * (in green) indicates the solvent peak.

The existence of supramolecular polymeric bQH-Zn and monomeric bQH-Zn_2_ complexes was also proved by the ^1^H-NMR study (Figure 4 and Figure S13). We first mixed the bQH solution (2.0 × 10^−2^ M in DMSO) with 2.0 equivalent of tetraethylammonium hydroxide. The imino proton at 12.37 ppm in the original ^1^H NMR spectrum of bQH disappeared while the protons in core benzene ring and quinoline rings exhibited relative upshifts, indicating the deprotonation of bQH. By adding the Zn(ClO_4_)_2_ gradually, a clear shifting of the protons were observed. When the Zn^2+^ was added in 1.0 equivalent, the bQH started to bind the Zn^2+^. The 1:1 stoichiometry leads the formation of polymeric bQH-Zn and it starts to precipitate in the solution (seen in the inset of Figure 4b), that is insoluble in common polar solvents including DMSO, DMF, methanol, chloroform, and water. The precipitate broadens the ^1^H-NMR spectrum, thus making it difficult to assign each proton accurately. Despite the difficulties in interpreting the peaks in the ^1^H-NMR spectrum, a clear shift of H^4^ and H^b^ to the downfield was observed, which could be assigned to the coordination of bQH to Zn^2+^ ion [58,59]. Upon further addition of Zn^2+^ to 2.0 equivalents, H^4^ and H^b^ exhibited further shifting to upfield, and the solution started to become clear again. The increment in Zn^2+^ content up to 4.0 equivalents, however, did not lead to any significant changes in chemical shifts of protons, but the sharpening of the spectrum was observed. This stationary and sharpened peaks in the ^1^H-NMR spectrum indicates that the formation of monomeric bQH-Zn_2_ complexes is predominant, which comes from the bQH-Zn after it disassembles by excess amount of Zn^2+^.

## 4. Conclusions

In summary, a bis-quinoline-based acyl hydrazone fluorescent probe (bQH) was designed and synthesized. Hydrazone has the advantage of enabling the facile synthesis of desired molecules at a low cost. The emissive nature of bQH was first observed on the surface of NBR gloves. For an in-depth understanding of bQH and its emissive nature, various experiments such as UV–Vis absorption, PL, NMR, ICP-OES spectroscopies, and EDS mapping were performed. It was discovered that bQH binds to Zn^2+^ ion selectively, making a bQH-Zn^2+^ complex that is emissive even in the solid-state. By addition of Zn^2+^ to bQH, the planarization of bQH led an increasing absorption band at 420 nm. The planarization further restricted the rotation of C–C bond between phenyl ring and acyl moiety, making the bQH-Zn^2+^ complex emissive. The binding modes of the bQH-Zn^2+^ complex were discovered to be composed of two parts with bQH:Zn^2+^ ratios of 1:1 and 1:2. The relatively weak binding strength of bQH, combined with the two binding sites, enables bQH to bind Zn^2+^ in two ways: Through metallo-suparmolecular polymers and monomeric complexes. These findings would encourage further development of fluorescent metal probes and their application in the research on solid-state emission.

## Supplementary Materials

The Supplementary Materials are available online at https://www.mdpi.com/1424-8220/20/3/600/s1.
