# Supplementary material for: Solid-State Emissive Metallo-Supramolecular Assemblies of Quinoline-Based Acyl Hydrazone
Source: Sensors (Basel). 2020 Jan 21;20(3):600. doi: 10.3390/s20030600 (PMC7037554; doi:10.3390/s20030600)
Supplement: Supplementary file 1 [file sensors-20-00600-s001.pdf]

Supplementary Material for

Solid-State Emissive Metallo-Supramolecular Assemblies of Quinoline-based Acyl Hydrazone

Hye Jin Cho [1,‡], *TaeWoo Kim* [1,‡], *Hyunwoo Kim* [1], *and Changsik Song* [1,*]

[1] Department of Chemistry, Sungkyunkwan University, 2066 Seobu-ro, Jangan-gu, Suwon-si, Gyeonggi-do, 16419, Republic of Korea.

E-mail: songcs@skku.edu

‡ These authors contribute equally to this work.

Contents:

Figure S1. Surface specific emission of bQH. p S2

Figure S2. EDS analysis of nitrile rubber surface. p S3

Table S1. Elements detected by EDS analysis. p S3

Figure S3. The metal-ion binding property of bQH. p S4

Figure S4. Effect of competitive metal ions. p S4

Figure S5. The anion effect on the metal binding capacity of bQH. p S5

Figure S6. The changes in absorption spectra of bQH by addition of CN^-. p S5

Figure S7. Absorption spectra of non-emissive bQH solutions with Zn^{2+}. p S6

Figure S8. Absorption spectra of emissive bQH solutions with Zn^{2+}. p S7

Figure S9. Absorption and emission spectra of mQH and bQH. p S8

Figure S10. The result of ICP-OES with the bQH solution dropped on NBR. p S9

Figure S11. Job's analysis of bQH. p S9

Figure S12. UV-Vis spectroscopy titration of bQH (1.0×10^{-6} M in DMSO) with $Zn(CN)_2$. p S10

Table S2. Binding constants of bQH toward the $Zn(CN)_2$. p S10

Table S3. FQY of QHs with different $Zn(CN)_2$ ratio. p S11

Figure S13. Partial ^{1}H-NMR (500 MHz) of bQH in Figure 4. p S12

Figures S14-15. ^{1}H and ^{13}C NMR spectra of bQH. p S13

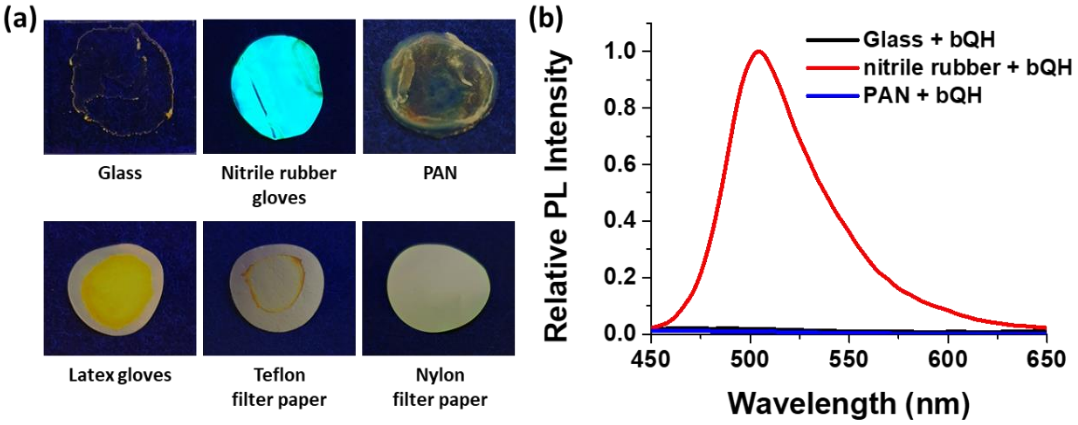

Figure S1. Surface specific emission of bQH. (a) Images of bQH on various substrates under 365 nm UV lamp, obtained by dropcasting (20 μL) the solution (2.0×10^{-2} M in DMSO) and dried under vacuum for 12 h. (b) PL spectra of bQH on three different substrates, which the intensity is shown relatively to that of bQH on nitrile rubber.

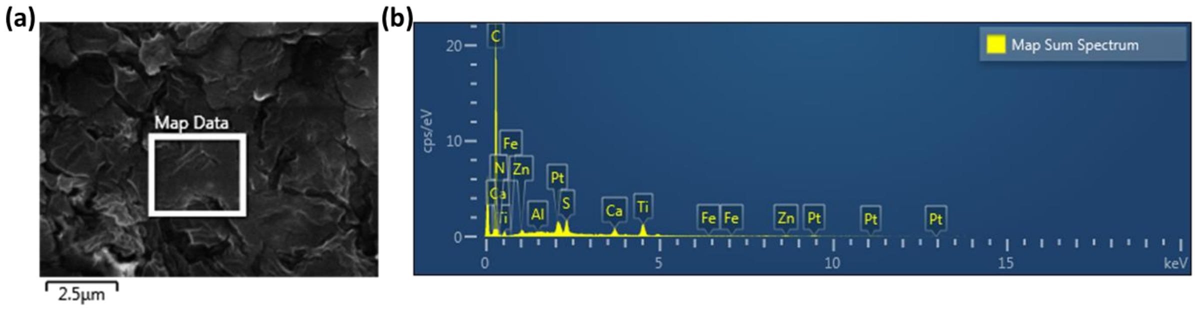

Figure S2. EDS analysis of nitrile rubber surface. (a) SEM image of nitrile rubber, (b) EDS spectrum of the area marked with the white box in (a).

Table S1. Elements detected by EDS analysis.

Element	Atomic number	Series	Wt%	Atomic %
Carbon	6	K series	82.87	92.47
Nitrogen	7	K series	3.44	3.29
Aluminium	13	K series	0.09	0.04
Sulfur	16	K series	2.65	1.11
Calcium	20	K series	2.35	0.79
Titanium	22	K series	7.16	2.00
Iron	26	K series	0.09	0.02
Zinc	30	L series	1.35	0.28

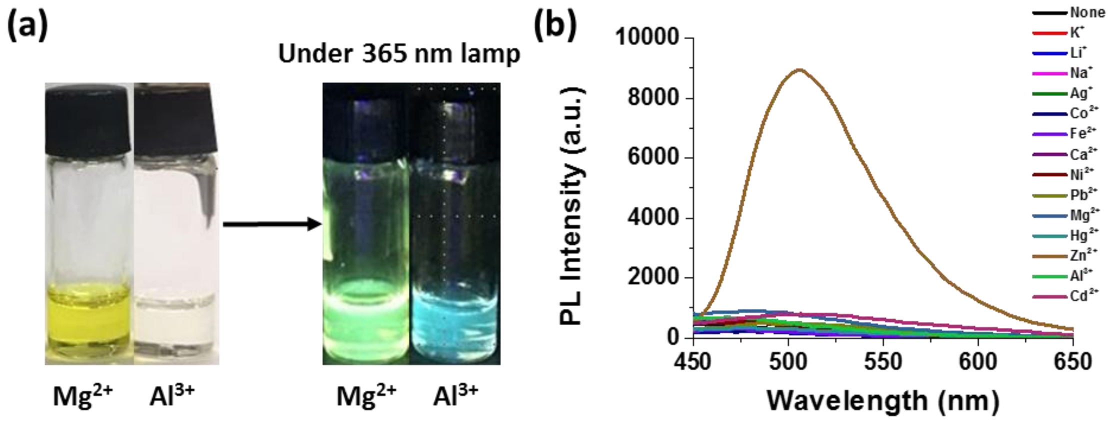

Figure S3. The metal-ion binding property of bQH. (a) Images of bQH solutions (ca. 5.0×10^{-3} M in DMSO) mixed with Mg^{2+} (left) and Al^{3+} (right), respectively. (b) PL spectra of bQH (5.0×10^{-6} M in DMSO) with 2 equiv. of metal perchlorates of stock solutions (5.0×10^{-3} M in DMSO). Measurement conditions: lamp voltage of 500 V, 10 nm slit width, and λ_{ex} = 350 nm.

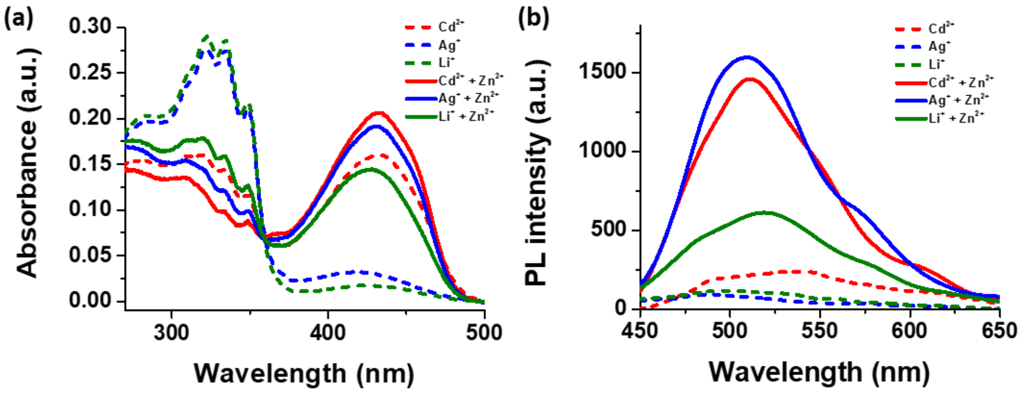

Figure S4. Effect of competitive metal ions. (a) Absorption and (b) emission spectra of bQH (5.0×10^{-6} M in DMSO) obtained by addition of 4.0 equiv. of TBA-CN followed by addition of 4.0 equiv. of each metal ions – Cd^{2+}, Ag^{+}, Li^{+}, respectively. Then, 4.0 equiv. of Zn^{2+} was added to the same solutions to obtain absorption and emission spectra. All metal ions were added by using the solutions of metal perchlorates in DMSO (2.0×10^{-5} M). Measurement conditions: lamp voltage of 500 V, 10 nm slit length, and λ_{ex} = 350 nm.

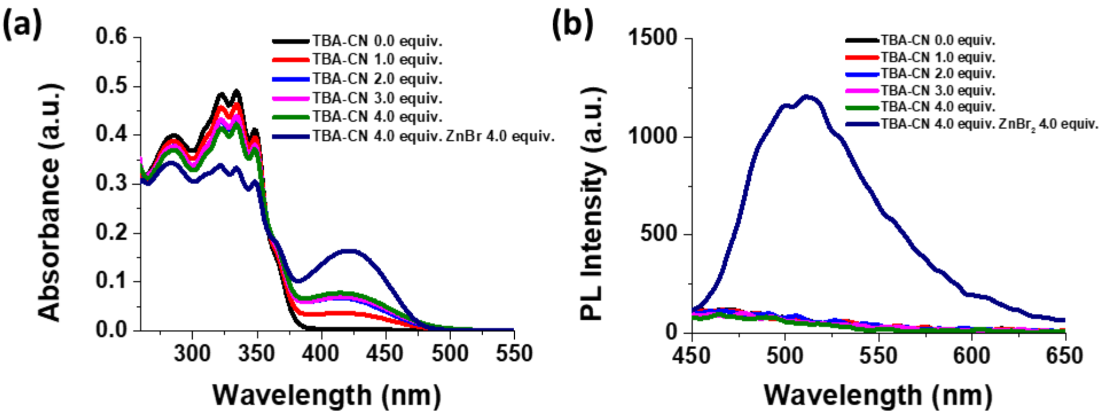

Figure S5. The anion effect on the metal binding capacity of bQH. (a) Absorption spectra of bQH (5.0×10^{-6} M in DMSO) with addition of CN^- from 0.0 to 4.0 equiv. using TBA-CN. (b) Emission spectra of corresponding samples of (a) and addition of Zn^{2+} by 4.0 equiv. to the bQH solution with 4.0 equiv. of TBA-CN. Measurement conditions: lamp voltage of 500 V, 5 nm slit width, and λ_{ex} = 350 nm.

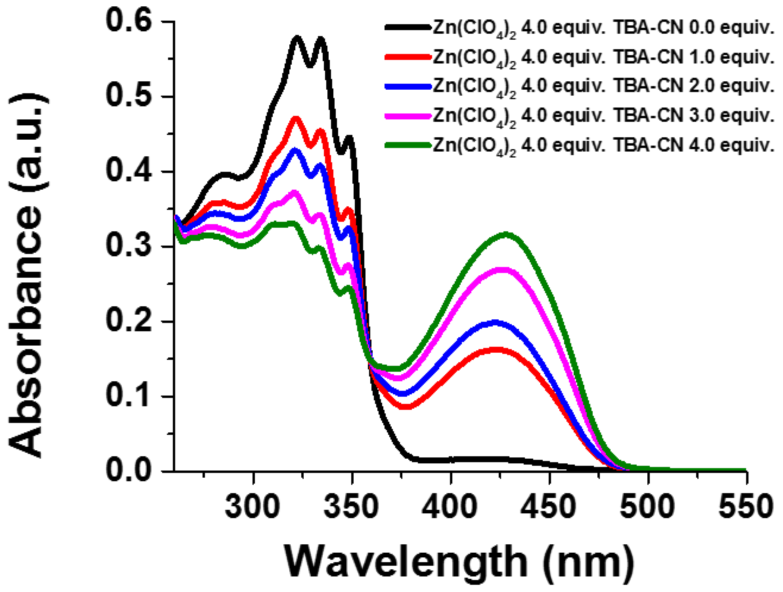

Figure S6. The changes in absorption spectra of bQH by addition of CN^-. The bQH solution (5.0×10^{-6} M in DMSO) was mixed with 4.0 equiv. of $Zn(ClO_4)_2$, by addition of CN^- from 0.0 to 4.0 equiv. using TBA-CN.

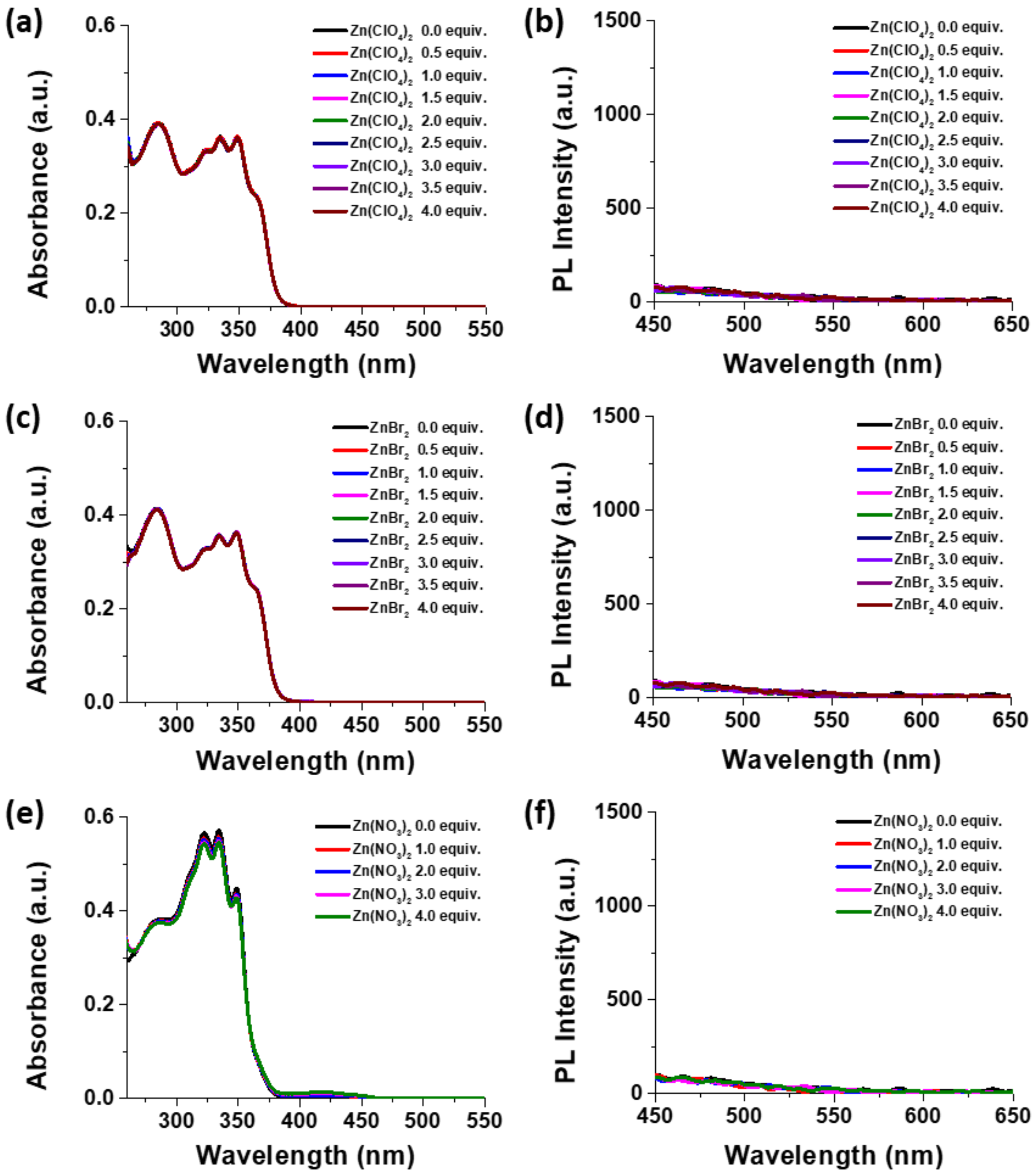

Figure S7. Absorption spectra of non-emissive bQH solutions with Zn^{2+}. The bQH solutions (5.0×10^{-6} M in DMSO) was added with various Zn sources. (a) $Zn(ClO_4)_2$, (c) $ZnBr_2$, (e) $Zn(NO_3)_2$ from 0.0 to 4.0 equiv. respectively. Emission spectra of corresponding samples added with (b) $Zn(ClO_4)_2$, (d) $ZnBr_2$, (f) $Zn(NO_3)_2$. Measurement conditions: lamp voltage of 500 V, 5 nm slit width, and λ_{ex} = 350 nm.

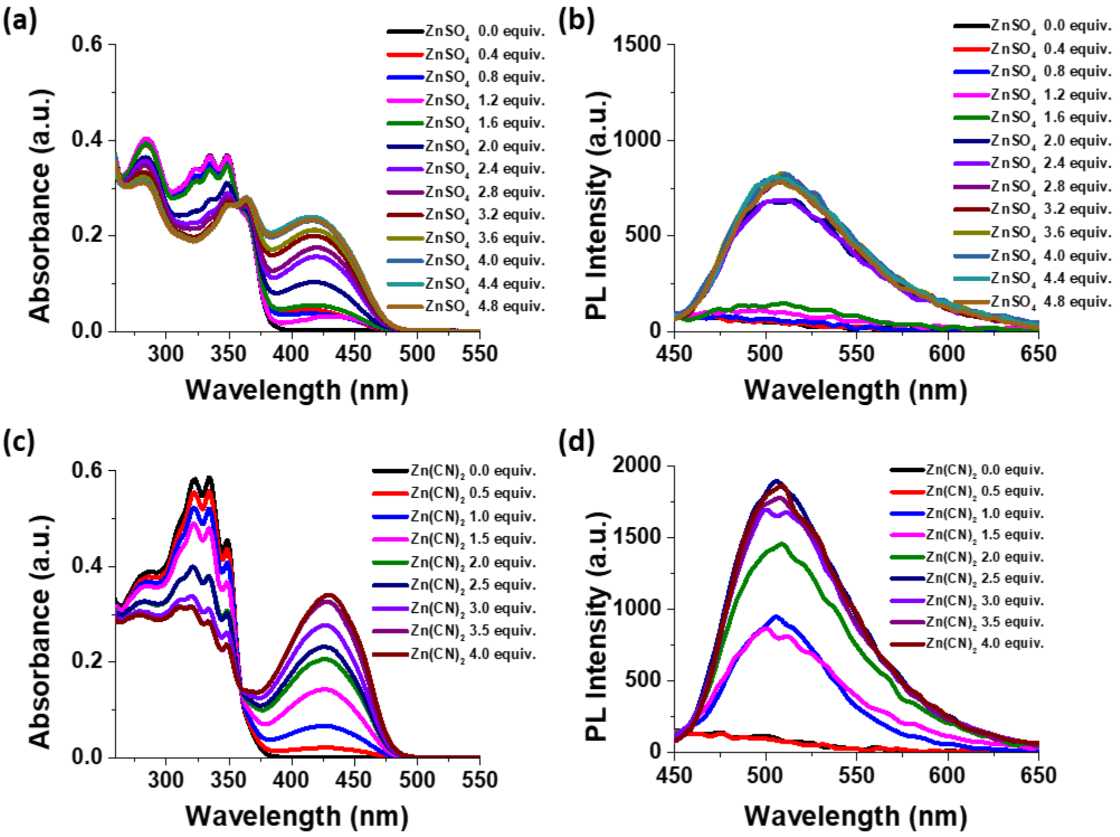

Figure S8. Absorption spectra of emissive bQH solutions with Zn^{2+}. Each spectrum was obtained from bQH solutions (5.0×10^{-6} M in DMSO), by addition of (a) $ZnSO_4$, (c) $Zn(CN)_2$ from 0.0 to 4.0 equiv. respectively. Emission spectra of corresponding samples added with (b) $ZnSO_4$, (d) $Zn(CN)_2$. Measurement conditions: lamp voltage of 500 V, 5 nm slit width, and λ_{ex} = 350 nm.

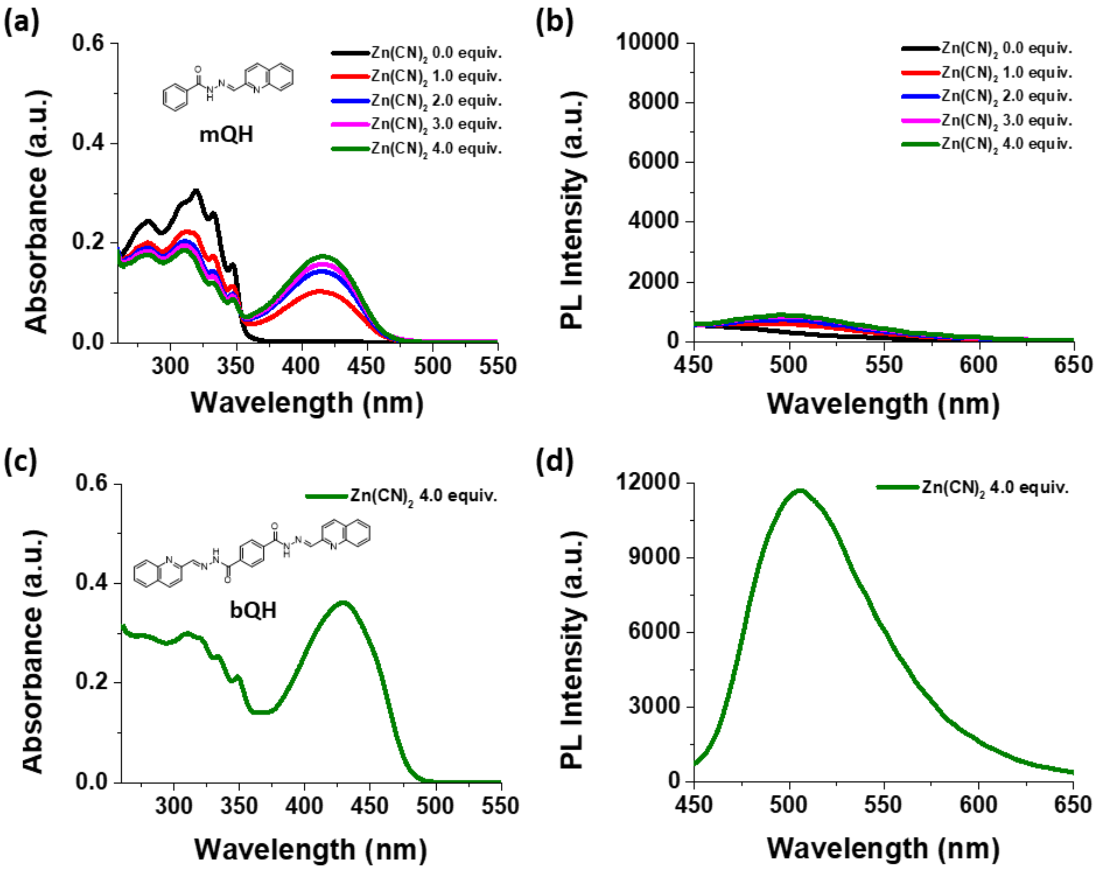

Figure S9. Absorption and emission spectra of mQH and bQH. Absorption spectra of (a) mQH and (c) bQH, and emission spectra of (b) mQH and (d) bQH were obtained from the bQH solutions (5.0×10^{-6} M in DMSO, respectively) by addition of $Zn(CN)_2$. Measurement conditions: lamp voltage of 500 V, 10 nm slit width, and λ_{ex} = 350 nm.

(a)

$$\text{Total concentration} = \text{Measured concentration} \times \frac{(\text{Total weight} - \text{Bottle weight})}{\text{Sample weight}}$$

(b)

Sample weight (g)	Bottle weight (g)	Total weight (g)	Measured concentration (mg/kg)	Total concentration (mg/kg)
0.4443	11.5559 g	45.7152 g	0.2647	20.3510

Figure S10. The result of ICP-OES with the bQH solution dropped on NBR. The 6.0×10^{-3} M bQH solution was prepared in DMSO. (a) The equation how to calculate the total concentration, and (b) the table of detailed results of ICP-OES measurement.

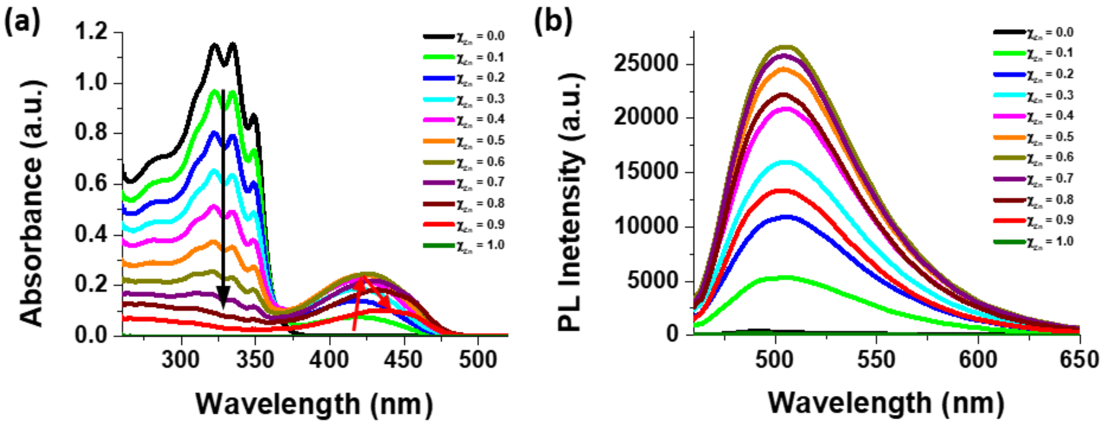

Figure S11. Job's analysis of bQH. (a) Absorption and (b) emission spectra of bQH with increasing ratio of Zn^{2+} ion ($[QH] + [Zn^{2+}] = 2.0 \times 10^{-5}$ M). Measurement conditions: lamp voltage of 500 V, 10 nm slit length, and $\lambda_{ex} = 430$ nm.

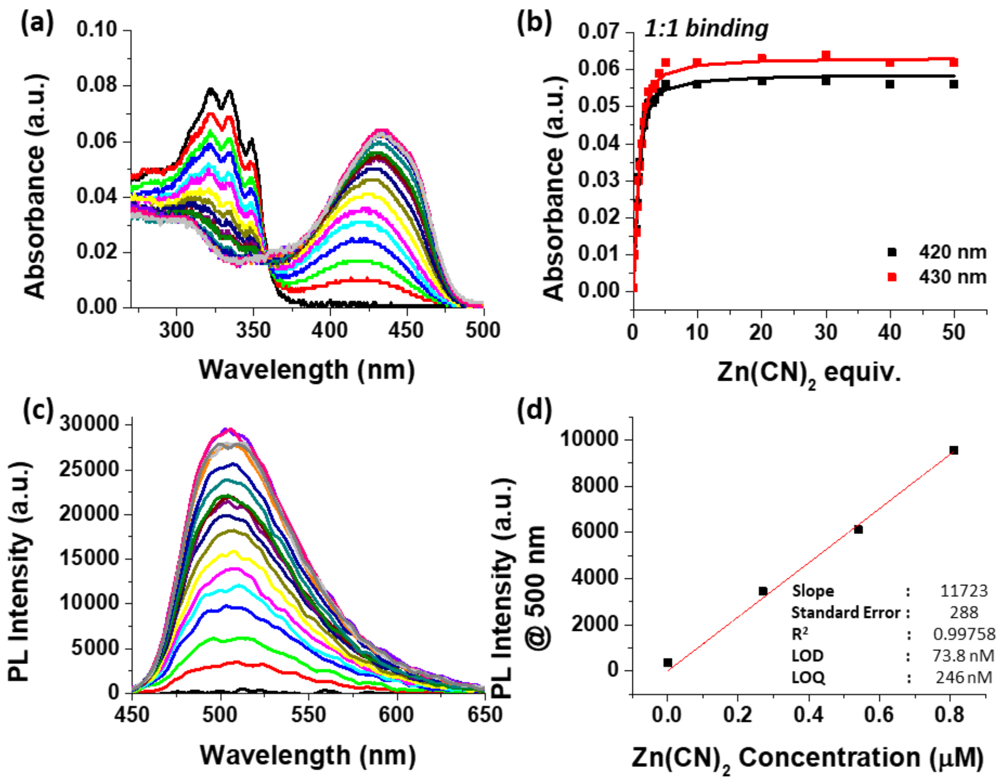

Figure S12. UV-Vis spectroscopy titration of bQH (1.0×10^{-6} M in DMSO) with $Zn(CN)_2$. (a) The obtained absorption spectra. (b) The fitting result with a non-linear regression method of the binding model 1:1. (c) The corresponding emission spectra of samples used in (a). Measurement conditions: lamp voltage of 700 V, 5 nm slit length, and λ_{ex} = 420 nm. (d) Determining the limit of detection (LOD) and limit of quantitation (LOQ) of bQH toward $Zn(CN)_2$ with the lowest 4 points from emission spectra of (c) at 500 nm.

Table S2. Binding constants of bQH toward the $Zn(CN)_2$.

Binding model	Standard error (10^{-3})	cov_{fit} (10^{-3})	K_1 (M^{-1})	K_2 (M^{-1})	α*
1:1	1.51	6.20	2.30×10^6	–	–
1:2	0.913	2.08	5.02×10^6	8.14×10^5	0.649

* The interaction parameter $\alpha = 4K_2/K_1$ with > 1 indicating positive cooperativity, α < 1 negative cooperativity, and α = 1 no cooperativity

Table S3. FQY of QHs with different $Zn(CN)_2$ ratio.

QH : Zn^{2+} ratio	Major compound	FQY (%) *
1:1	mQH-Zn	0.37
1:5	mQH-Zn_2	0.30
1:1	bQH-Zn	0.82
1:5	bQH-Zn_2	1.06

* Using quinine sulfate as a standard, λ_{ex} = 380 nm.

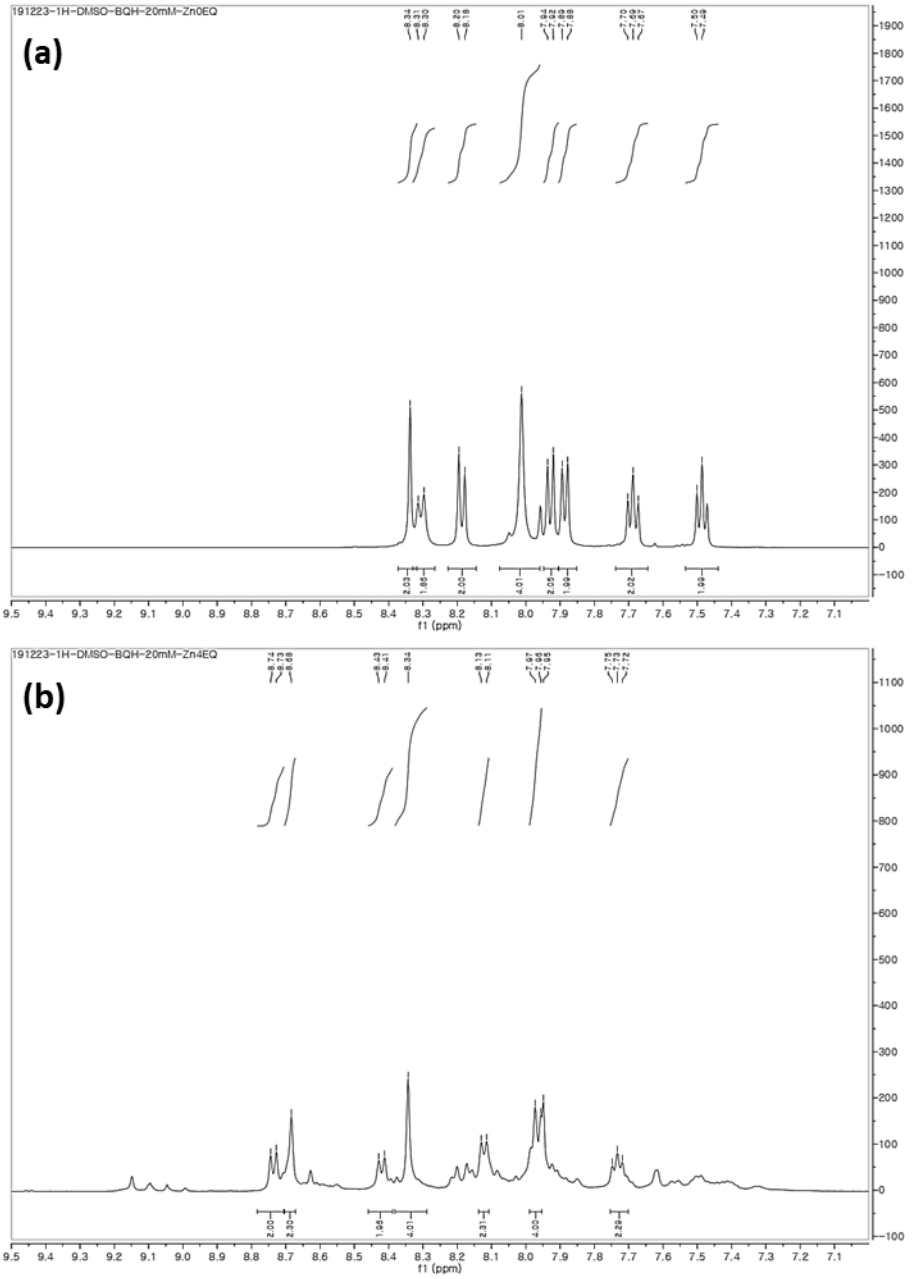

Figure S13. Partial ^{1}H-NMR (500 MHz) of bQH in Figure 4. (a) bQH + 2 equiv. TEA-OH, and (b) bQH + 2 equiv. TEA-OH with 4 equiv. $Zn(ClO_4)_2$.

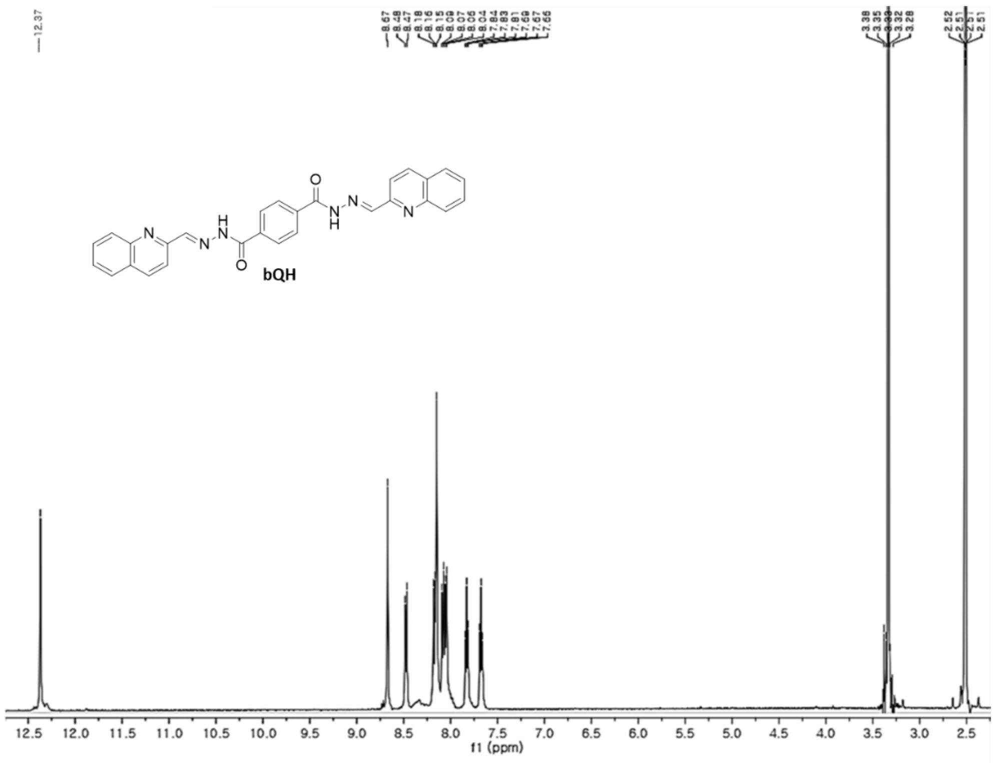

Figures S14. ^{1}H NMR spectrum of bQH (500 MHz, DMSO-d_6).

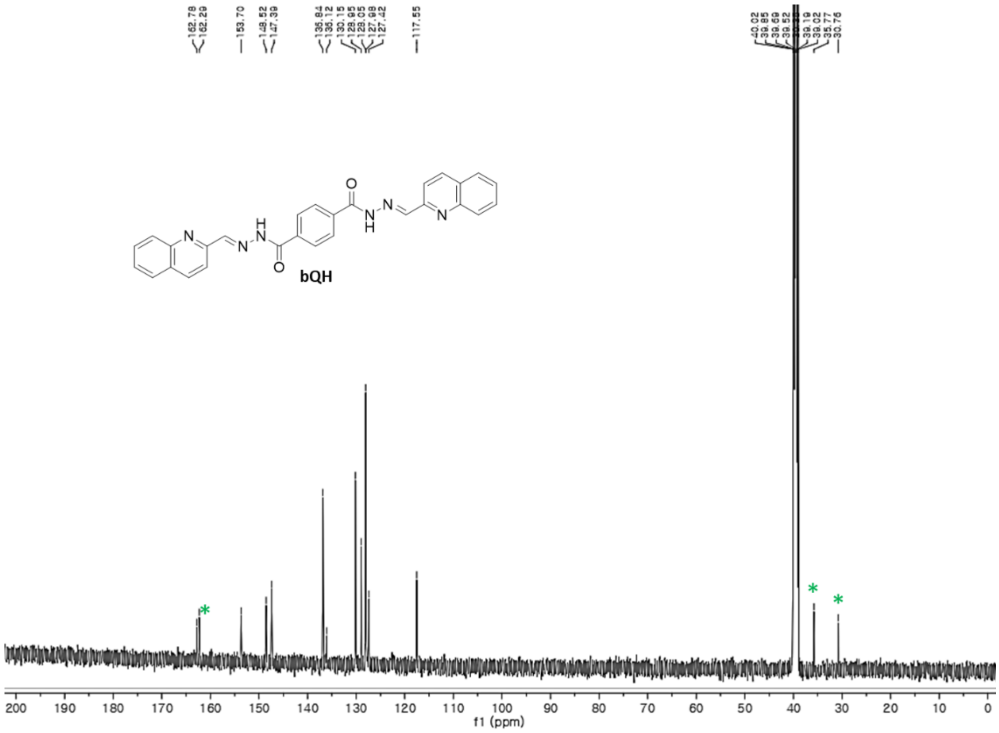

Figures S15. ^{13}C NMR spectrum of bQH (125 MHz, DMSO-d_6). The * refers to solvent peaks.
